# Cervical Carcinoma Manifesting as Progressive Bilateral Visual Loss

**DOI:** 10.1155/2014/757261

**Published:** 2014-08-06

**Authors:** O'Sam Shibeeb, Paul Athanasiov, Sumu Simon, Jagjit Gilhotra

**Affiliations:** ^1^Ophthalmic Research Laboratories, South Australian Institute of Ophthalmology, Hanson Institute Centre for Neurological Diseases, University of Adelaide, Frome Road, Adelaide, SA 5000, Australia; ^2^Department of Ophthalmology and Visual Sciences, Royal Adelaide Hospital, Frome Road, Adelaide, SA 5000, Australia

## Abstract

We report a patient with bilateral choroidal metastasis from disseminated cervical squamous cell carcinoma. A 52-year-old woman presented with progressive bilateral visual loss due to choroidal masses in both eyes. The fundus examination revealed posterior serous retinal detachment in both eyes associated with creamy choroidal lesions. A thorough systemic work-up revealed choroidal metastasis from a squamous cell carcinoma of the cervix. This case highlights the importance of a thorough systemic evaluation in patients with choroidal tumours.

## 1. Introduction

Choroidal metastasis is the most common intraocular malignancy in adults [1]. The posterior choroid is highly susceptible to disseminated tumour cells compared to the other uveal structures due to its vascular nature; hence many choroidal metastases are bilateral [2]. The majority of choroidal metastases arise from breast malignancies in women and lung malignancies in men; less common sites of primary malignancy include the gastrointestinal tract, prostate, and kidney [1]. Choroidal metastasis secondary to cervical squamous cell carcinoma (SCC) is extremely rare [1, 3], with only 3 cases previously reported [2, 4, 5]. The current case is unique in the fact that it resulted in bilateral loss of vision and that there was no prior knowledge of the primary malignancy.

## 2. Case Report

**Figure 1:**
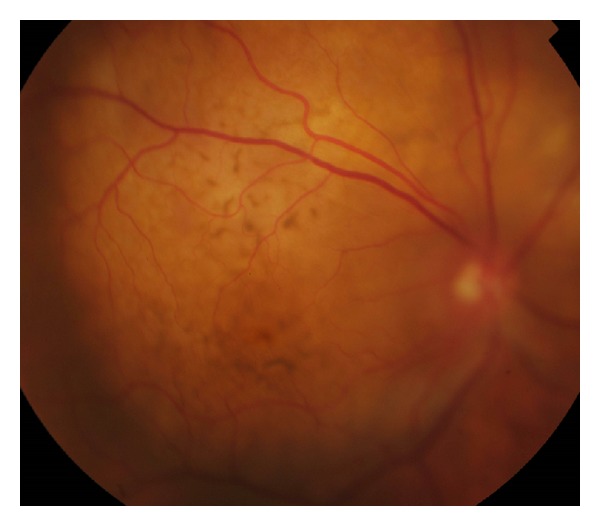
Fundus photograph of the right posterior pole (focused anteriorly on the elevated retina): multiple cream-coloured choroidal lesions with fine retinal pigment epithelial mottling.

**Figure 2:**
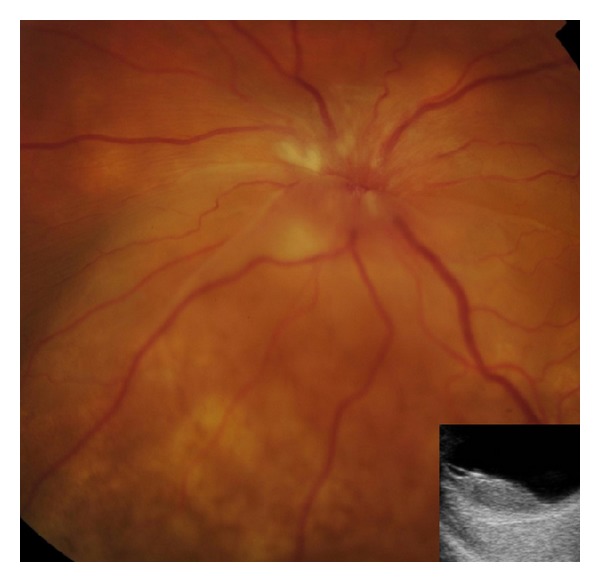
Fundus photograph of the left posterior pole: multiple creamy-coloured choroidal lesions with fine retinal pigment epithelial mottling and optic disc oedema with inferior margins obscured by the exudative retinal detachment. Associated with nerve fibre layer ischaemia. Inset: B-scan ultrasound demonstrating elevated choroidal lesions and associated serous retinal detachment.

A 52-year-old Caucasian woman presented with a three-month history of progressive, painless, bilateral visual loss. The only significant medical history was that of regular cigarette smoking. Visual acuity was “counting fingers” (at one metre) in each eye. Anterior segment examination was unremarkable. Fundus examination demonstrated posterior exudative retinal detachment in the right eye associated with creamy, ill-defined choroidal lesions and retinal pigment epithelial mottling (Figure 1). Fundoscopy of the left eye similarly demonstrated elevated, creamy choroidal lesions with bullous exudative retinal detachment associated with optic disc oedema and never fibre layer ischaemia. B-scan ultrasonography of the left eye revealed a dome-shaped lesion of intermediate internal reflectivity without extrascleral extension (Figure 2). Further history and examination failed to identify a possible primary carcinoma. Computerized tomography (CT) imaging revealed an invasive cervical tumour with widespread metastatic disease to the liver and long bones. Cervical biopsy demonstrated a large-cell type squamous cell carcinoma and liver biopsy confirmed metastatic spread. Palliative external beam radiotherapy was undertaken over two weeks to both eyes and the visual acuity improved to 6/36 in the right eye and to 6/60 in the left eye. The patient died three months after presentation.

## 3. Discussion

The breast and lung are the primary sites of malignancy in approximately 70% of cases of choroidal metastasis [3]. A literature search found three previous cases of uterocervical carcinoma metastatized to the choroid: two cases of adenocarcinoma and one case of SCC [3–5]. Each of these cases had prior history of systemic malignancy.

The current case demonstrates a rare and devastating cause of bilateral, progressive vision loss due to metastatic squamous cell carcinoma of the cervix without prior diagnosis of the primary cancer. SCC is a particularly aggressive form of cervical cancer with five-year survival rate of 20–25% [2]. It spreads by direct extension and lymphatic dissemination; however, in advanced stages, it may develop into haematogenous metastatic disease. The common sites for distant metastasis include extra-pelvic lymph nodes, liver, lung, and bone [2].

External beam radiotherapy can be used to improve visual function in this setting, as with other cases of choroidal metastasis [4]. Combined radiotherapy and chemotherapy (usually cisplatin-based) may improve life expectancy. One study demonstrated systemic improvements using bevacizumab chemotherapy; off-label intraocular injections of bevacizumab (Avastin) may therefore be another option for improving visual function in patients with choroidal metastasis of squamous cell carcinoma [6].

In summary, SCC can, in rare situations, metastasize to the choroid; however, the fundus features noted in this case should trigger the differential diagnosis of choroidal metastasis with appropriate systemic investigation, including pelvic imaging.
